# Supplementary material for: Risk Factors for Venous Thromboembolism in Severe COVID-19: A Study-Level Meta-Analysis of 21 Studies
Source: Int J Environ Res Public Health. 2021 Dec 8;18(24):12944. doi: 10.3390/ijerph182412944 (PMC8700787; doi:10.3390/ijerph182412944)

SUPPLEMENT

Supplemental Figure S1. Funnel plot of studies reporting D-dimer as prognostic factor for VTE (Egger's regression test for asymmetry p=0.50)

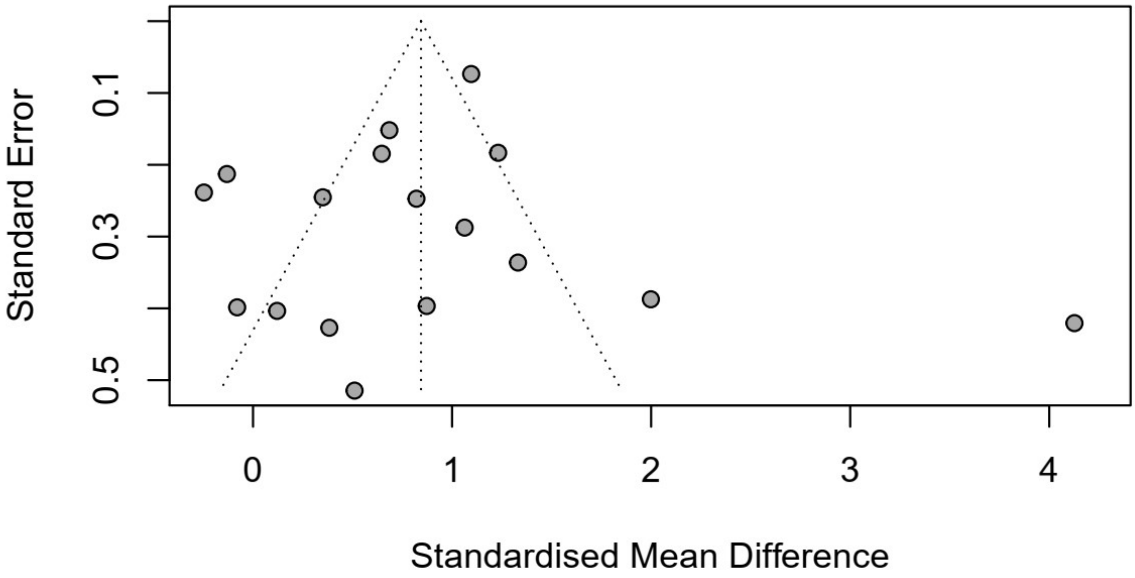

Supplemental Figure S2. Funnel plot of studies reporting C-reactive protein as prognostic factor for VTE (Egger's regression test for asymmetry p=0.54)

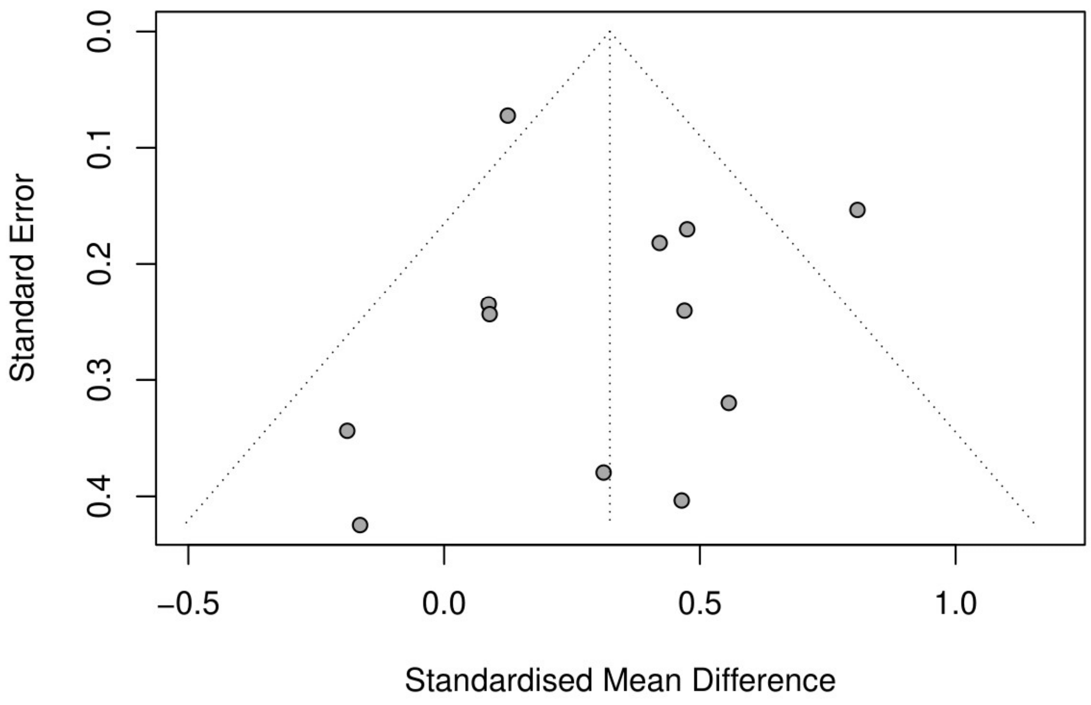

Supplemental Figure S3. Funnel plot of studies reporting platelet level as prognostic factor for VTE (Egger's regression test for asymmetry p=0.86)

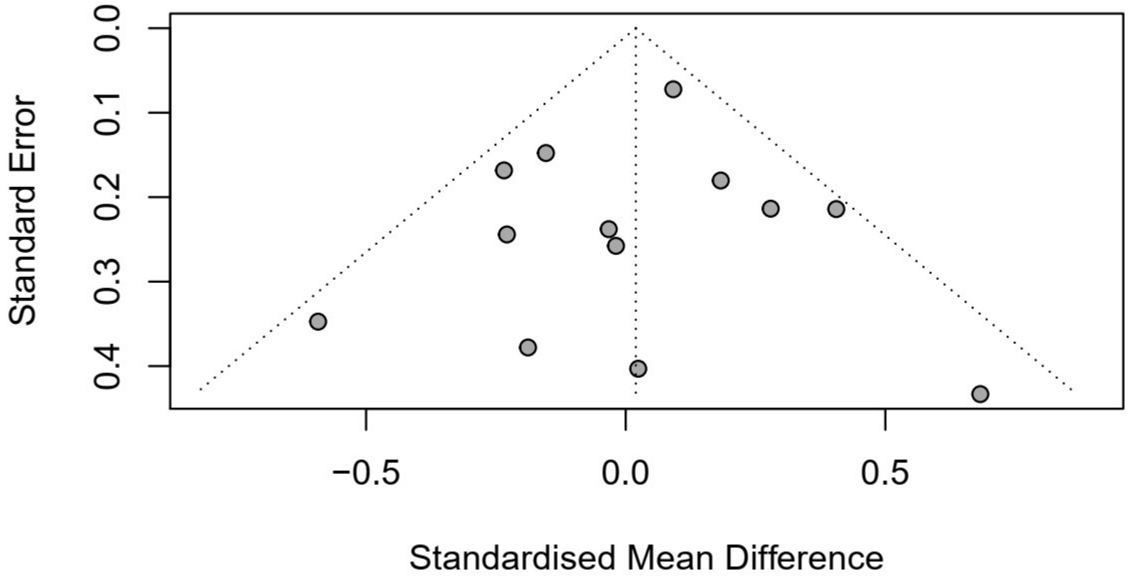

Supplemental Figure S4. Funnel plot of studies reporting age as prognostic factor for VTE (Egger's regression test for asymmetry p=0.80)

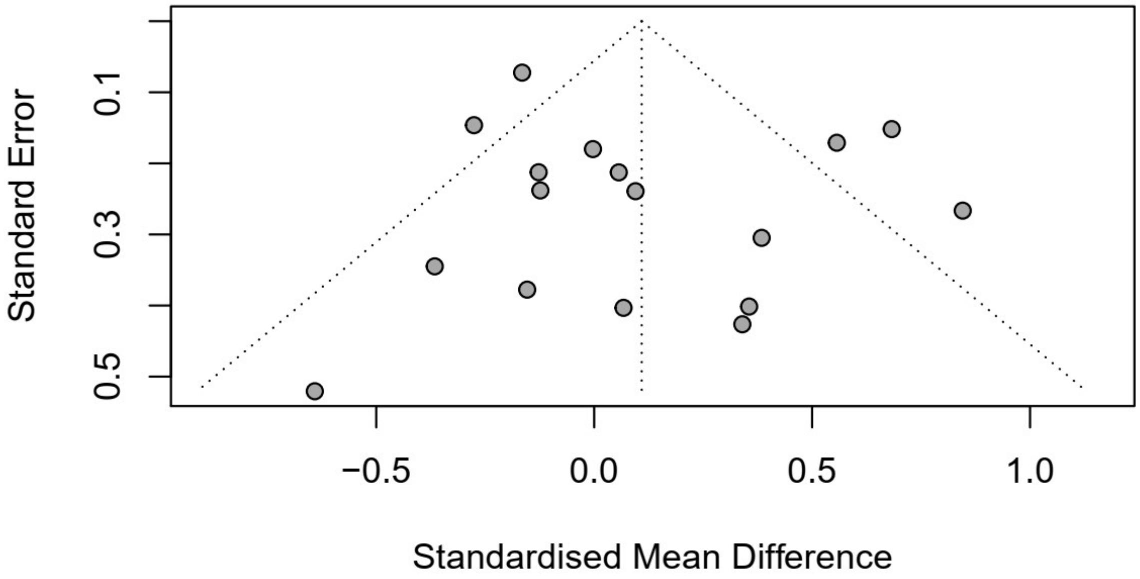

Supplemental Figure S5. Funnel plot of studies reporting body mass index as prognostic factor for VTE (Egger's regression test for asymmetry p=0.71)

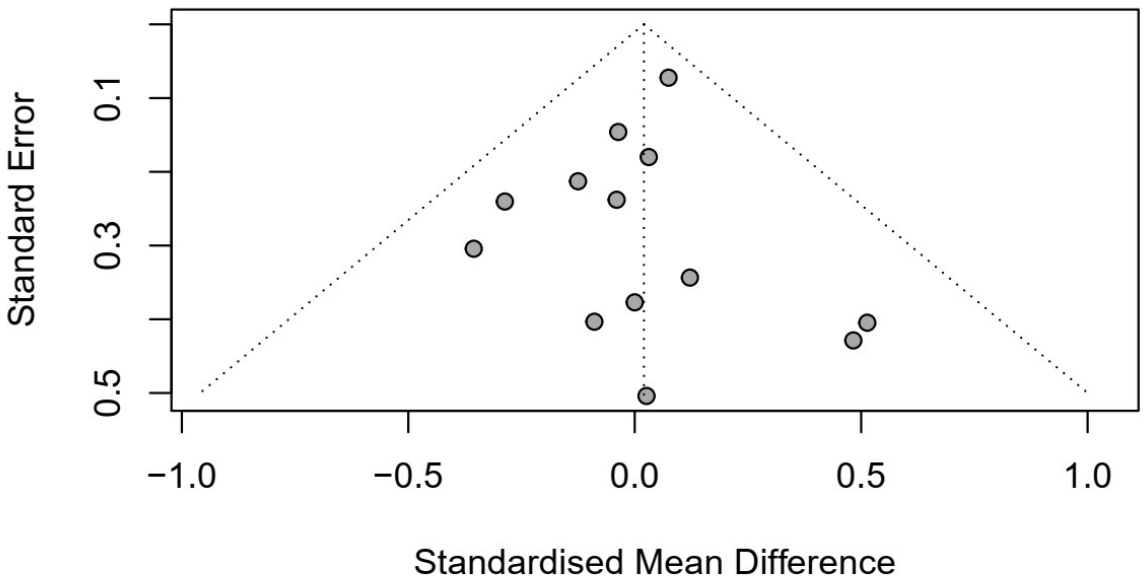

Supplemental Figure S6. Funnel plot of studies reporting fibrinogen level as prognostic factor for VTE (Egger's regression test for asymmetry p=0.07)

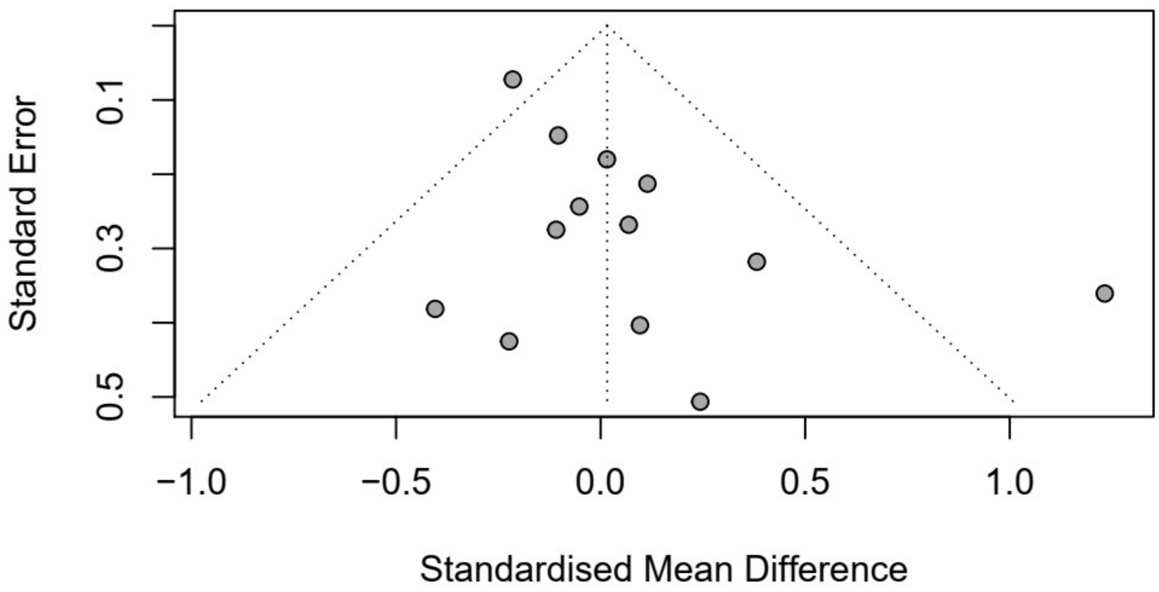

Supplement: Supplementary file 1 [file ijerph-18-12944-s001.zip › ijerph-1477541-supplementary.pdf]
